# Combined high-intensity interval training and green tea supplementation enhance metabolic and antioxidant status in response to acute exercise in overweight women

**DOI:** 10.1186/s12576-020-00756-z

**Published:** 2020-06-25

**Authors:** Elham Ghasemi, Mohammad Esmaeil Afzalpour, Shila Nayebifar

**Affiliations:** 1 Department of Sport Sciences, Faculty of Literature and Humanities, University of Zabol, Zabol, Iran; 2 Department of Exercise physiology, Faculty of Sport Sciences, University of Birjand, Birjand, Iran; 3 Department of Sport Sciences, Faculty of Educational Sciences and Psychology, University of Sistan and Baluchestan, Zahedan, Iran

**Keywords:** HIIT, Catechins, SRIT1, PGC-1α, CAT

## Abstract

Thirty sedentary overweight women were randomly assigned to three groups (*n* = 10), including HIIT + green tea, HIIT + placebo and green tea. The training program included 3 sessions/week HIIT while the supplement consuming groups took 3 * 500 mg of green tea tablets/day for 10 weeks. Results indicated that 10 weeks of HIIT and green tea meaningfully pronounced baseline serum levels of SIRT1 (*P* ≤ 0.0001), PGC-1α (*P* ≤ 0.0001) and CAT (*P* ≤ 0.0001). In addition, significant increase was observed in three indicators in HIIT + green tea group in comparison with two other research groups. Further, the responses of SIRT1 (*P* ≤ 0.01) and CAT (*P* ≤ 0.002) increased significantly to second acute exercise in all three groups. The combination of HIIT and green tea consumption may induce increasing SIRT1 and CAT in response to acute exercise and can improve antioxidant system, body composition and VO_2_ max results rather than green tea and training alone, in young sedentary overweight women.

## Background

Aging and inactive lifestyle are associated with higher risks of obesity and overweight that can cause several diseases. Identification of biomarkers helps clinicians’ control and treats these diseases. Among the important factors involved in controlling metabolic disorders and preventing obesity are Sirtuin-1 (SIRT1) and Peroxisome Proliferator-Activated Receptor Gamma Co-activator 1-Alpha (PGC-1α) [1]. Although reports of reduced SIRT1 relate to intracellular SIRT1 (mRNA or protein), Kumar et al. [2] first reported that SIRT1 was detectable in the serum. In other report, SIRT1 was measured by various methods, including Western blot, ELISA, and surface plasmon resonance, with good correlation with each method, confirming that SIRT1 is a serum protein [3]. This report was surprising, as SIRT1 had originally been described only as a nuclear protein, however recent reports have demonstrated that SIRT1 can shuttle between the nucleus and cytoplasm [3]. Therefore, SIRT1 is potentially present in the extracellular component [4, 5]. Although the main source of circulating SIRT1 is not known research results indicate that the negative metabolic effects of obesity could be related, at least in part, to the reduced levels of SIRT1 in the blood [6, 7]. Moreover, what regulates circulating SIRT1 vs. tissue SIRT1 is still unknown. So far, there is no evidence that plasma or serum SIRT1 is associated with cell damage. As for this, these indicators were measured in the blood in the current study.

SIRT1 is a NAD+-dependent protein deacetylase to combat oxidative stress and control homeostasis, which is also known as the elixir of life and longevity factor [8]. After SIRT1 was activated, the function of the PGC-1α, as is a key regulator of gluconeogenesis and fatty acid oxidation which cooperates with Hepatocyte Nuclear Factor 4 (HNF4α), increases [9].

Also, SIRT1 can increase the antioxidant enzymes expression such as catalase (CAT) and superoxide dismutase of the Superoxide dismutase (SOD) by activating the Forkhead box O3 (FOXO3) transcription factors in the nucleus to combat free radicals [10]. According to the results of studies, antioxidant defense factors in obese individuals are at lower levels [11, 12], for this reason, these individuals are more prone to oxidative damages. According to evidence, the impact of high intensity interval training (HIIT) on SIRT1, PGC-1α and CAT levels have been reported previously. These trainings are considered as one of the obesity prevention and treatment strategies in addition to a cost-effective protocol for increasing SIRT1, PGC-1α, CAT, weight loss and fat loss in obese individuals [13–16].

On the other hand, nowadays, it is thought that dieting with antioxidant property can play an important role in preventing the risk of obesity-related diseases [17]. There are five major polyphenol called catechins in green tea, the most important of which is epigallocatechin gallate (EGCG) that suppresses oxidative stress and obesity-related diseases via the SIRT1/PGC-1α signaling pathway [18]. EGCG and green tea catechins leads to increase the function of PGC-1α, decrease malondialdehyde, hydroperoxides, and increase the activity of antioxidant enzymes by activating SIRT1, thereby energy homeostasis will be regulated [19].

Growing evidence suggests HIIT as vital method in exercise programs; however, interestingly, despite this evidence, few randomized trials have directly evaluated the effect of acute and chronic HIIT exercises on SIRT1, PGC-1α, and CAT indicators and the interactive effects of HIIT and green tea supplementation, in inactive overweight individuals. The researchers aimed to assess the impact of 10-week high-intensity interval training and green tea supplementation on response to acute exercise and baseline serum levels of SIRT1, PGC-1α and CAT in overweight women.

## Methods

### Participants

Thirty overweight young women (aged 20–30 years and body mass index > 25 kg/m^2^) volunteered to participate in this randomized, placebo-controlled study. The Ethics Committee of Birjand University of Medical Sciences (Iran) has approved the study proposal while the Iranian Registry of Clinical Trials code is (http://www.irct.ir; IRCT2015121425524N1). Following a verbal and written explanation of the nature and risks involved in the study, written, informed consent was obtained from all volunteers then subjects were randomly assigned to three groups of ten, according to age, body mass index (BMI), namely, HIIT + green tea, HIIT + placebo and green tea. Inclusion criteria were age 20–30 years, overweight BMI ≥ 25 kg/m^2^, not meeting the current physical activity guidelines, non-smokers and willing to participate in an exercise intervention and no history of physical activity prior the study. Exclusion criteria included diagnosis of chronic diseases (type 2 diabetes, cardiovascular, renal, etc.), musculoskeletal problems, and taking medications or dietary supplements known to affect the primary outcomes of the study.

None of the participants was excluded from the statistical population considering these criteria. The calories burned during exercise/activities can be calculated using following formula [20]: calories burned (CB) = duration (in min) * MET * 3.5 * weight (in kg)/200.

Also, dietary assessment has been estimated through 24-h recall questionnaire method 1 week prior the start of the intervention and the final week. During the protocol, since the food regimen was influential on the research variables, all of the participants had the same diet (university restaurant). It should be noted, the participants were also asked to avoid the consumption of black tea, coffee, beer, juice, any pill or drug supplementation and performing intense physical activity. Then macro, micronutrients and calories calculated according to the instructions of Dorosti Food Processor software. All subjects were weighed barefoot and with minimal clothing using a digital scale (TCM, China) both before and after the protocol. Body mass index (BMI) was calculated as the weight (kg) divided by the square of the height (m^2^). Percent body fat was determined using Jackson–Pollock 3-Site Skin fold procedure with caliper ((SH5020, England) [21] according to standard protocol. Also, VO_2_ max was assessed on treadmill (Cosmos T, Cos10199, h-p-150 model) performing Maximum Bruce Test and the following formula [22]:

$$V{\text{O}}_{{2\max }} = (4.38 \times {\text{total exercised time}}) - 3.9.$$

#### The supplement intake procedure

According to safety doses in previous studies [16, 23, 24] participants in supplement groups received 1500 mg green tea tablets (produced by Iran Dineh Co., Tehran, Iran) daily, whereas the HIIT + placebo group received starch powder tablets (produced by Iran Dineh Co., Tehran, Iran) in the same manner, for 10 weeks, 7 days/week, 3 times/day, 2 h after their main meals. Furthermore, we used green tea tablets with a certain amount of Catechin presented from the company. Each tablet contained 500 mg green tea consisted of 300 mg of Catechin (each 500 mg tablet contained ~ 294 mg EGCG, 74 mg EGC, 20.7 mg EC, 51.2 mg ECG, 58.1 mg Caffeine). During the work, the consumption of the tablets was pursued regularly using social networks and SMS.

#### The HIIT protocol

**Fig. 1:**
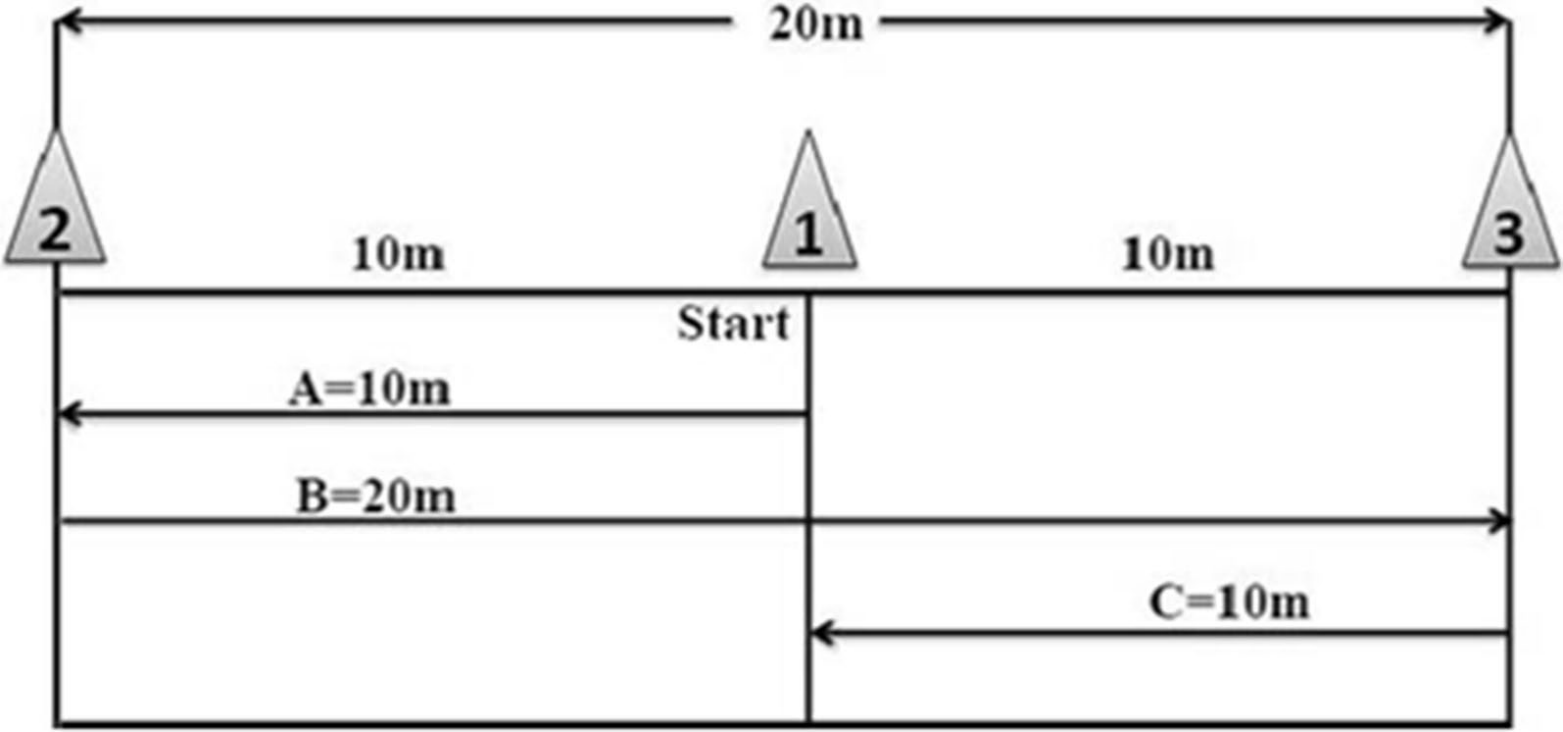
Course outline showing distance and direction taken by participants, during the 30-s HIIT protocol

The exercise protocol was taken from 40-m maximal shuttle run, which is a valid test for the assessment of anaerobic function. During this activity, each participant totally ran and returned a 20 m route at her maximum speed in 30 s (Fig. 1) [25]. The exercise protocol was performed three times per week for 10 weeks as shown in Table 1. The exercise was conducted at 90% maximum heart rate (age—220) intensity which was controlled by a Telemetry (Polar, Finland).

**Table 1:** The training program in 10 weeks

	Weeks	Time of activity (s)	Time of active rest (s)	Frequency	Time of protocol (min)	The total duration of activity (main activity, warm-up the cooling) (min)
Protocol 40-m shuttle run	First and second	30	30	4	4	24
	Third and fourth	30	30	5	5	25
	Fifth and sixth	30	30	6	6	26
	Seventh and eighth	30	30	7	7	27
	Ninth and tenth	30	30	8	8	28

#### The acute exercise protocol

The acute exercise trial was started with a brief warm-up (cycling at 50 W for 5 min) followed immediately by the acute protocol (4 × 30-s all-out cycling at a constant load corresponding to 0.075 kg/kg body mass, i.e., Wingate tests) separated by 4 min of active rest on an electronically braked ergo [26]. Heart rate was collected during exercise using telemetry (Pollar, Finland). Changes in plasma volume were calculated as described by Dill and Costill [27]. Then, the indices were corrected for changes in plasma volume.

#### Biochemical analyzing

One day before and 72 h after the last training session, subjects arrived in the laboratory after 10–12 h fasting. Blood samples (~ 5 mL) were drawn from a superficial vein in the forearm, using standard vein puncture techniques immediately before and after the acute exercise on ergometer while subjects were in their follicular phase of menstruation (from the first to midpoint of the phase) [28]. Blood samples were put in blood tubes containing EDTA and then spun at 2000×*g* for 10 min in a refrigerated (4 °C) centrifuge (MPW-350R, Med. Instruments, Poland). The serum was stored at—80 °C for future analysis. SIRT1 and PGC-1α levels were analyzed using Cusabio China-made kit (respectively, sensitivity < 39% ng/mL and intra-assay CV: < 7%; and sensitivity < 31/25 pg/mL and intra-assay CV: < 8%). Catalase was also assayed using the German ZellBio kit with a sensitivity < 0.5 μg/mL and intra-assay CV < 6.3%. All the measurements were performed using ELISA method.

#### The statistical analyzing

Data were analyzed using SPSS (Statistical Package for the Social Sciences) version 19 software (SPSS Inc, Chicago, IL, USA). The data are expressed as mean ± standard deviation. After assessing the normality and non-normality of data by Shapiro–Wilk test, paired-samples *t* test was conducted for assessing pretest–posttest within-group variations (changes in weight, BFP, BMI and VO_2_ max), while changes in the serum SIRT1, PGC-1α and CAT mean were assessed using two-way repeated measures and ANOVA, where the within factor was acute exercise (pre-exercise vs. post-exercise) and the between factor was training status (HIIT + green tea vs. HIIT + placebo vs. green tea). For the data that showed a significant interaction effect, Tukey post hoc test and one-way ANOVA, were used to locate the differences.

## Results

The demographic characteristics of the participants in this study are listed in Table 2. According to the results of one-way ANOVA, there is no significant difference in pre-test values of demographic indices. Using one-way ANOVA on scores of pre-tests and the difference of post-test and pre-test scores showed calories burned (energy expenditure during exercise) increased in training–green tea and training–placebo; however, there was no significant difference in dietary energy intake in all three groups.

**Table 2:** Characteristics of anthropometric and body composition in the various groups

Variable	Training–green tea	Training–placebo	Green tea	*P*-values	*F*
Age (years)	22.47 ± 3.32	23.58 ± 2.23	21.06± 2.65	0.21	1.67
Weight (kg)	70.56 ± 6.19	72.18 ± 3.51	73.45 ± 8.44	0.68	0.39
BMI (kg/m^2^)	27.15 ± 1.47	27.32 ± 1.27	28.03 ± 1.04	0.54	0.62
Body fat (%)	34.12 ± 1.80	33.57 ± 1.39	34.28 ± 1.38	0.43	0.80
VO_2_ max (ml/kg/min)	24.5 ± 2.32	23.65 ± 3.31	24.05 ± 3.16	0.69	0.76

Values are mean ± SD

According to the results of two-way repeated measures on SIRT1, PGC-1α and CAT indices, there are significant differences between these indices statistically at both research stages (measurement stages) and between groups, while the interaction between time and group is also significant (*P* ≤ 0.05).

**Fig. 2:**
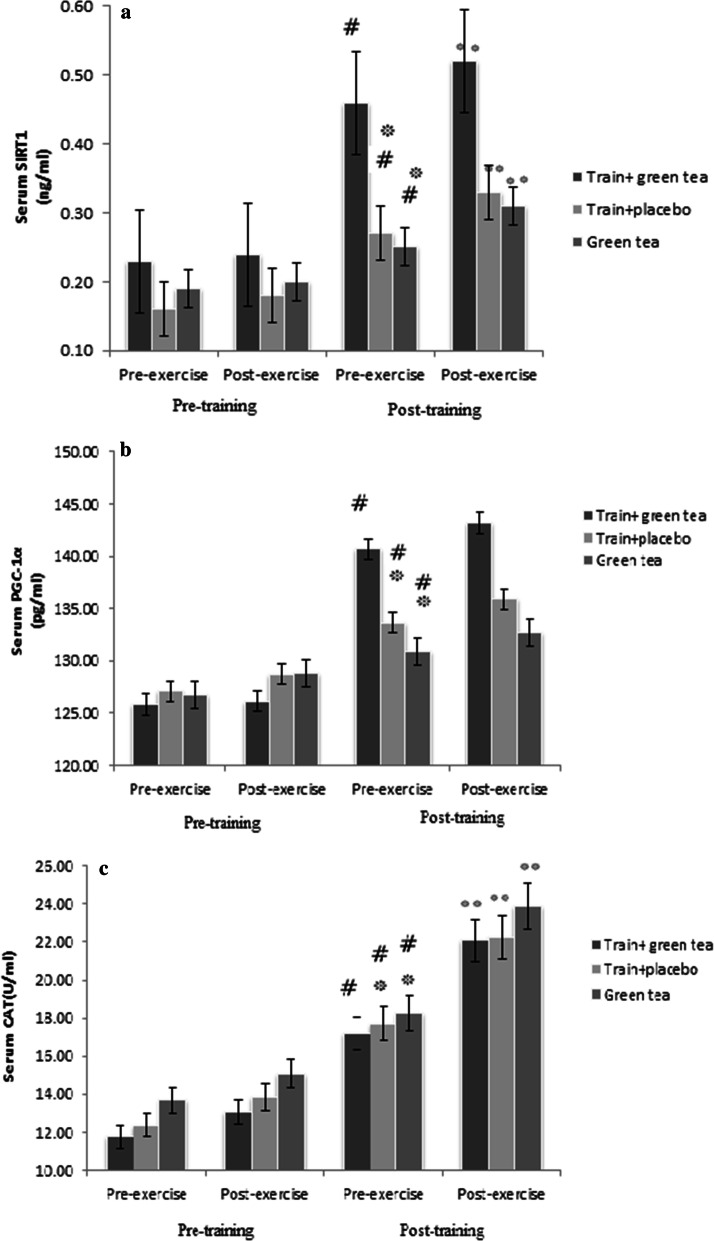
**a** The effects of training and green tea on the serum SIRT1 in response to an acute exercise session. **b** The effects of training and green tea on the serum PGC-1α in response to an acute exercise session. **c** The effects of training and green tea on the serum CAT in response to an acute exercise session. ^#^Significant difference with baseline (first) *P* ≤ 0.05, *significant difference with train + green tea group *P* ≤ 0.05, **significant difference in response to acute exercise *P* ≤ 0.05

Following the 10-week HIIT and green tea intervention, a significant increase was observed in the baseline levels of SIRT1 indices (*P* < 0.0003, *P* < 0.01, *P* < 0.006, respectively) (Fig. 2a), PGC-1α (*P* < 0.0001, *P* < 0.03, *P* < 0.002, respectively) (Fig. 2b) and CAT (*P* < 0.0001, *P* < 0.02 and *P* < 0.0001, respectively) (Fig. 2c) in the groups of HIIT + green tea, HIIT + placebo and green tea.

According to the results of Tukey’s post hoc test for intergroup comparisons, the mean changes in baseline serum levels of SIRT1, PGC-1α and CAT in the HIIT + green tea group was significantly higher than the HIIT + placebo group (*P* < 0.003, *P* < 0.01 and *P* < 0.04, respectively) and the green tea group (*P* < 0.0001, *P* < 0.03, and *P* < 0.006, respectively).

Moreover, serum SIRT1 (*P* < 0.01, *P* < 0.01 and *P* < 0.02, respectively) and CAT (*P* < 0.001, *P* < 0.01 and *P* < 0.03, respectively) showed significant increase in response to second acute exercise in groups of HIIT + green tea, HIIT + placebo and green tea. The one-way ANOVA showed significant difference between three groups in serum SIRT1 group in response to second acute exercise (*P* < 0.01). Results of post hoc test showed significant increase in HIIT + green tea group in comparison with HIIT + placebo (*P* < 0.01) and green tea (*P* < 0.02) (Fig. 2).

Furthermore, according to the results of *t* test, the indices of weight (*P* < 0.001, *P* < 0.03, and *P* < 0.04, respectively), BMI (*P* < 0.03, *P* < 0.02 and *P* < 0.04, respectively) and BFP (*P* < 0.001, *P* ≪ 0.001 and *P* < 0.04, respectively) were decreased significantly compared to pre-test in the three groups of HIIT + green tea, HIIT + placebo and green tea, respectively, whereas VO_2_ max increased significantly only in the HIIT + green tea, HIIT + placebo groups (*P* < 0.007).

Moreover, according to Table 3 and the results of ANOVA, there was a significant difference between the mean changes in weight, BFP, BMI and VO_2_ max between the three groups (*P* < 0.001); also, according to the results of Tukey’s post hoc test, mean changes in BFP (*P* < 0.001 and *P* < 0.001, respectively), BMI (*P* < 0.001 and *P* < 0.0001, respectively), weight (*P* < 0.001 and *P* < 0.001, respectively) and VO_2_ max (*P* < 0.02 and *P* < 0.03, respectively) in the HIIT + green tea group significantly more than in the groups of HIIT + placebo and green tea alone (Table 4).

**Table 3:** The results of ANOVA to compare body fat, BMI, weight and VO_2_ max between groups in the different stages of intervention

Variables	Groups	Before training	After training	*P*-values
Body fat (%)	Training–green tea	34.12 ± 1.80	27.12 ± 1.45	> 0.001^†^
	Training–placebo	33.57 ± 1.39	29.57 ± 1.90	
	Green tea	34.28 ± 1.38	32.42 ± 1.27	
BMI (kg/m^2^)	Training–green tea	27.15 ± 1.47	25.31 ± 1.24	>0.001^†^
	Training–placebo	27.32 ± 1.27	26.45 ± 1.49	
	Green tea	28.03 ± 1.04	27.46 ± 1.26	
Weight (kg)	Training–green tea	70.56 ± 6.19	65.83 ± 6.27	>0.001^†^
	Training–placebo	72.18 ± 3.51	69.88 ± 3.79	
	Green tea	73.45 ± 8.44	71.95 ± 6.37	
VO_2_ max (mL/kg/min)	Training–green tea	24.5 ± 2.32	28.42 ± 2.23	>0.001^†^
	Training–placebo	23.65 ± 3.31	25.47 ± 2.91	
	Green tea	24.05 ± 3.16	25.8 ± 3.38	

Values are mean ± SD. ^†^Significant difference at *P* < 0.05

**Table 4:** The results of Tukey post hoc test to paired comparison of body fat, BMI, VO_2_ max, weight

Groups comparisons	Mean difference ± SE	Tukey (*P*)
Training + green tea vs. training + placebo	− 0.97 ± 0.34	*0.001
Training + green tea vs. green tea	− 1.17 ± 0.64	*0.0001
Training + placebo vs. green tea	− 0.19 ± 0.01	0.99
Training + green tea vs. training + placebo	− 3.01 ± 0.76	*0.001
Training + green tea vs. green tea	− 5.14 ± 0.35	*0.001
Training + placebo vs. green tea	− 2.14 ± 0.05	*0.001
Training + green tea vs. training + placebo	− 2.42 ± 0.55	*0.001
Training + green tea vs. green tea	− 3.22 ± 0.32	*0.001
Training + placebo vs. green tea	− 0.80 ± 0.03	*0.047
Training + green tea vs. training + placebo	− 2.19 ± 0.43	*0.02
Training + green tea vs. green tea	− 2.16 ±0.09	*0.03
Training + placebo vs. green tea	− 0.43 ± 0.05	*0.032

*SE* standard error. *Significant difference at *P* < 0.05

## Discussion and conclusion

The aim of this study was twofold. First, we sought to examine the effect of 10 weeks of HIIT (40-m maximal Shuttle run) induced changes in serum SIRT1, PGC-1α and CAT levels following an acute high-intensity interval exercise challenge performed at the same absolute intensities as those undertaken prior to training. Second, since green tea is an antioxidant plant, we aimed to determine if green tea consumption together with repeated acute exercise exposure (i.e., training) or alone, affects the serum these markers response to an acute exercise stress. The important findings of the study were that in overweight females, a 10-week HIIT regimen together with green tea could attenuate serum SIRT1 and CAT more than two other groups in response to acute exercise.

Very few studies have dealt with the combined effect of exercises and green tea consumption on antioxidant indices. Consistent with the findings of the present study based on increase SIRT1, PGC-1α and CAT serum levels after 10 weeks HIIT, Little et al. [10] reported that performing HIIT for 2 weeks in 7 young women caused to increased nuclear expression of PGC-1α and SIRT1 levels but no change in PGC-1α protein content [29].

Also, some studies have also reported inconsistent results. For example, 6-week HIIT and endurance training was found to have no significant effect on the response of SIRT1 and PGC-1α to an acute exercise [30].

One study showed an increase in PGC-1α and no change in SIRT1 levels following a 4-week HIIT with 170% aerobic power [31]. Also, Hassan et al. [32] concluded that there was a decrease in CAT levels following a 30-week HIIT among 30 active people with the same protocol as in the present study.

It seems that the results of this study are inconsistent with the above studies due to differences in type and exercise protocols and physical fitness levels of subjects. In both the mentioned papers, the subjects were male and healthy and the protocol duration was 4 and 8 weeks, while inactive overweight female subjects were selected and the training period was 10 weeks in the present study.

Because SIRT1 protects cells under conditions of oxidative stress and is regulated, itself, by oxidative stress [33], we hypothesized that SIRT1 function is influenced by dietary intake of antioxidants flavonoid (green tea) and training. Treatment with flavonoid (green tea catechins) leads to increased mitochondrial biogenesis and increased energy expenditure in mice, possibly by SIRT1-mediated increase in PGC-1α activity [19, 34]. It is also possible that the effects on BMI are caused by an influence of SIRT1 on appetite and energy intake, since SIRT1 is highly expressed in brain [4, 6]. Previous studies showed that weight loss induces an increase in tissue and circulating SIRT1 levels in obese patients [4, 5, 13]. Thus, SIRT1 tissue expression and activity is influenced by the availability of energy suggesting that SIRT1 could have a role in the regulation of normal energy balance. In this regard, in the present study, energy expenditure exercise increased after the intervention, while no change was observed in dietary energy intake.

Accordingly, serum SIRT1 levels and fat mass are inversely regulated with SIRT1 concentrations being increased in a catabolic condition and decreased in conditions of extreme BMIs [8, 35].

In addition, the present study confirmed a negative strong relationship between SIRT1 and PGC-1α levels and fat percentage, BMI and weight, and it can be concluded that improvement of body composition can be due to increased levels of SIRT1 and PGC-1α in overweight women.

It is not clear how SIRT1 and PGC-1α influence BMI or Fat percentage, but potential mechanisms include a repressive effect on PPARc, central effects through satiety, increased energy expenditure, or effects by modification of Clock genes [4–6]. One limitation of the present study was lack of measurement of upstream and downstream factors of SIRT1 and PGC1α such as AMPK, calmodulin and PPARa, PPARy or measuring them via biopsies in humans to determine the exact signaling pathway of HIIT and green tea supplement in controlling weight. Further studies are needed to investigate the relation between SIRT1 and PGC-1α, and human body composition traits.

Also, according to our results, daily consumption of 1500 mg green tea for 10 weeks leads to increase the levels of SIRT1, PGC-1α and CAT and decrease BFP, BMI and body weight significantly. Catechin and EGCG in green tea are considered as the most important substances affecting SIRT1 and PGC-1α. Also, catechins leads to decrease adipocyte differentiation and proliferation, and decrease the expression of genes involved in lipogenesis, increase adiponectin levels, decrease leptin and prevent obesity and weight gain by activating SIRT1 reported [17–19]. However, one limitation of the present study was lack of measurement of plasma catechin concentration and more specific studies are needed in this regard.

It seems catechins (EGCG) existing in green tea by inhibiting phospholipase A2 and acetylcoa carboxylase, prevents lipogenesis and causes increasing fat oxidation and antioxidant capacity even at rest [17–19] and when consumption of this supplement combined with moderate to intensive physical activity, this increase will be more tangible [16, 36]. Also, researchers concluded that HIIT through phosphate and calcium-dependent pathways and the activity of AMPK lead to increase SIRT1 and CAT gene expression [15]. On the other hand, calcium release following muscle contraction leads to activate calmodulin, calcineurin and calmodulin kinase and increase SIRT1 and PGC-1α gene expression and activate PPARs in different tissues of the body and increase differentiation and reduce adipocyte size, lipid oxidation and fatty acids in the mitochondria and increase capillary network and mitochondrial density and finally increase the maximum oxygen consumption and reduce BFP [15, 28, 37].

### Novelty statement

Green tea ingested with HIIT for 10 weeks as an effective method has metabolic consequences such as significant increase in SIRT1, PGC1α and CAT levels that have not been previously considered. More importantly, this design can reduce undesirable effects of obesity and overweight by increasing the levels of STRT1 and CAT in response to acute exercise.

### Practical application

Although green tea consumption (at a dose of 1500 mg per day) causes increasing SIRT1, PGC1α and CAT and subsequently improving body composition in overweight women, it seems that its consumption along with regular HIIT (with intensity above 90% HR_max_ during 10 weeks) has bigger favorable impact on these indicators without any adverse cardiovascular effect. However, measuring upstream and downstream factors of SIRT1 such as AMPK, calmodulin and PPAR to determine the exact signaling pathway of HIIT and green tea supplement in controlling weight remain to be determined.

## Data Availability

Not applicable.

## Abbreviations

HNF4α: Hepatocyte Nuclear Factor 4
SOD: Superoxide dismutase
FOXO3: Forkhead box O3
VO_2_ max: Maximum oxygen consumption
BMI: Body mass index
EGCG: Epigallocatechin gallate
BFP: Percent body fat
PGC1α: Peroxisome Proliferator-Activated Receptor Gamma Co-activator 1-Alpha
CAT: Catalase
HR_max_: Maximum heart rate
SIRT1: Sirtuin-1
AMPK: AMP-activated protein kinase
HIIT: High-intensity interval training
PPAR: Peroxisome Proliferator-Activated Receptor
